# Down-regulation of S100P induces apoptosis in endometrial epithelial cell during GnRH antagonist protocol

**DOI:** 10.1186/s12958-021-00787-0

**Published:** 2021-07-02

**Authors:** Dan Zhang, Mi Han, Mingjuan Zhou, Mengyu Liu, Yan Li, Bufang Xu, Aijun Zhang

**Affiliations:** 1 Department of Obstetrics and Gynecology, Ruijin Hospital, Shanghai Jiao Tong University School of Medicine, 197 Ruijin 2nd Road, Shanghai, 200025 China; 2 Department of Histo-Embryology, Genetics and Developmental Biology, Shanghai Jiaotong University, School of Medicine; Shanghai Key Laboratory of Reproductive Medicine, 280 South Chongqing Road, Shanghai, 200025 China

**Keywords:** GnRH antagonist, S100P, Apoptosis, Endometrial receptivity

## Abstract

**Background:**

The gonadotropin-releasing hormone (GnRH) antagonist protocol for in vitro fertilization (IVF) often leads to lower pregnancy rates compared to the GnRH agonist protocol. Decreased endometrial receptivity is one reason for the lower success rate, but the mechanisms underlying this phenomenon remain poorly understood. The S100 calcium protein P (S100P) is a biomarker for endometrial receptivity. Both GnRH antagonist and S100P are involved in mediating cell apoptosis. However, the involvement of S100P in reduced endometrial receptivity during the GnRH antagonist protocol remains unclear.

**Methods:**

Endometrial tissue was collected at the time of implantation window from patients undergoing the GnRH agonist (GnRH-a) or GnRH antagonist (GnRH-ant) protocols, as well as from patients on their natural cycles. Endometrial cell apoptosis and expression levels of S100P, HOXA10, Bax, and Bcl-2 were assessed. Ishikawa cells were cultured to evaluate the effects that GnRH antagonist exposure or S100P up- or down- regulation had on apoptosis.

**Results:**

Endometrial tissue from patients in the GnRH-ant group showed elevated apoptosis and decreased expression of the anti-apoptotic marker Bcl-2. In addition, endometrial expression of S100P was significantly reduced in the GnRH-ant group, and expression of HOXA10 was lower. Immunofluorescence colocalization analysis revealed that S100P was mainly distributed in the epithelium. In vitro experiments showed that knockdown of S100P in Ishikawa cells induced apoptosis, decreased expression of Bcl-2, while overexpression of S100P caused the opposite effects and decreased expression of Bax. Furthermore, endometrial epithelial cells exposed to GnRH antagonist expressed lower levels of S100P and Bcl-2, increased expression of Bax, and had higher rates of apoptosis. The increased apoptosis induced by GnRH antagonist treatment could be rescued by overexpression of S100P.

**Conclusions:**

We found that GnRH antagonist treatment induced endometrial epithelial cell apoptosis by down-regulating S100P, which was detrimental to endometrial receptivity. These results further define a mechanistic role for S100P in contributing to endometrial apoptosis during GnRH antagonist treatment, and suggest that S100P is a potential clinical target to improve the success of IVF using the GnRH antagonist protocol.

## Background

The gonadotropin-releasing hormone (GnRH) antagonist (GnRH-ant) protocol for reproductive assistance has several advantages compared to the classical GnRH agonist (GnRH-a) long protocol, including shorter treatment duration, lower gonadotropin requirements, and a potential reduction in the incidence of severe ovarian hyperstimulation syndrome. However, although there are conflicting reports comparing these two protocols, most studies report that the GnRH-ant protocol is associated with a lower pregnancy rate than the GnRH-a long protocol for fresh embryo transfer cycles [1, 2]. We have previously found that reduced dosing of GnRH-ant during the protocol could improve the success of pregnancy facilitated by the GnRH-ant protocol [3]. Endometrial receptivity, not embryo quality, is considered to be the primary factor contributing to the difference in pregnancy outcomes, as no significant differences in clinical outcomes we found in frozen-thawed embryo transfer cycles between patients treated with GnRH-a and those treated with GnRH-ant protocol [4].

Endometrial receptivity is one of the pivotal prerequisites for successful embryo implantation, and involves the coordinated activity of many factors, including estrogen and progesterone. During IVF, GnRH-ant inhibits the luteinizing hormone (LH) peak by competitive binding with GnRH to GnRH receptors on the surface of hypothalamus and adenohypophysis cells. However, the GnRH receptor is also expressed in endometrial tissue, and GnRH-ant could act antagonize GnRH signaling in endometrial cells [5–7], which may be detrimental to endometrial receptivity. GnRH-ant treatment can alter the migration of endometrial epithelial cells [8], and can increase abundance of uterine natural killer (uNK) cell numbers and elevate perforin expression [9]. However, the precise mechanisms by which GnRH-ant impacts endometrial receptivity remain to be clarified.

Treatment with GnRH-ant is known to induce apoptosis in a variety of cell types [10–12]. We have previously reported that apoptosis in endometrial cells is higher among patients treated with GnRH-ant than in a control group [9]. S100P is a small molecular weight calcium binding protein that is involved in cancer occurrence and progression, and is highly up-regulated during the mid-secretory phase of normal fertile endometrium [13]. S100P is also involved in cellular apoptosis [14]. Therefore, we hypothesized that S100P may be involved in mediating the elevated endometrial cell apoptosis seen in patients undergoing the GnRH-ant protocol.

The GnRH-ant protocol is the preferred method for IVF procedures, and it is of great importance to determine ways to improve the endometrial receptivity for patients treated with the GnRH-ant protocol. In this study, we evaluated the role of S100P in endometrial receptivity related to the GnRH antagonist protocol, and we determined the effects of S100P on endometrial cell apoptosis induced by GnRH-ant treatment.

## Methods

### Patients and sample collection

This study included 26 patients who received in vitro fertilization/intracytoplasmic sperm injection (IVF/ICSI) using GnRH antagonist protocol (*N* = 15) or GnRH agonist protocol (*N* = 11) for the first time and gave up fresh embryo-transfer due to personal reasons (such as altering pregnancy plan, intending to collect more embryos, feeling uncomfortable or other unexpected situation) or embryo reasons at the Reproductive Medical Center of Shanghai Jiaotong University affiliated Ruijin hospital from January 2019 to May 2021, and another 15 patients in their natural cycle were recruited as control. The inclusion criteria included: 25 to 35 years old, regular ovulatory cycles with every 27 to 32 days, normal serum levels of follicle stimulating hormone (FSH < 10 mIU/mL) and luteinizing hormone (LH < 10 mIU/mL) on day 2 to 3 of the menstrual cycle; the exclusive criteria included: presence of abnormal ovarian or/and endometrial ultrasonographic features, use of contraceptive drugs or intrauterine devices within the last 6 months, with endometriosis or polycystic ovary syndrome, and the number of oocytes obtained over than 15.

Clinical characteristics of this cohort, including ovarian stimulation information and the methods of endometrial specimen collection have been described previously [9]. In brief, GnRH antagonist protocol: rFSH 150 to 300 U (Gonal-F, Merck Serono S.A., Switzerland) stimulation was initiated on day 2 of the menstrual cycle. The gonadotropin dose was initiated and adjusted by doctors according to the personal status and ovarian response of the patients. Patients received 0.25 mg Cetrorelix acetate per day (Cetrotide, Merck Serono SA, Switzerland) from stimulating day 6 and continued daily until the trigger day. GnRH agonist long protocol: Patients received a standard full dose of 3.75 mg triptorelin (Decapeptyl, Ipsen, Signes, France) in the early follicular phase. After 28 to 30 days when pituitary downregulation was confirmed (indicated by serum FSH < 5 U/L, LH < 5 U/L and E2 levels < 50 pg/mL and endometrial thickness < 5 mm), the patients were administered Gonal-F 150 to 300 U daily until oocyte maturation was triggered. Natural control: Patients had their follicles reviewed every 1 to 2 days since after menstrual day 10.

Trigger and luteal phase supports: For the patients in ovulation cycles, if three or more follicles reached a mean diameter of 17 mm, 5000 to 7000 IU of human chorionic gonadotropin (hCG, Lizhu, Zhuhai, China) was administered intramuscularly. Oocyte retrieval was performed 35 to 36 h after hCG injection by transvaginal ultrasound-guided single-lumen needle aspiration. Luteal phase support with 600 mg of micronized progesterone (Crinone, Merck Serono S.A., Switzerland) was initiated on day 1 after oocyte retrieval till the biopsy day. For patients of natural control group, if one or more follicles reached a mean diameter of 17 mm, 5000 IU of hCG (Lizhu, Zhuhai, China) was administered intramuscularly.

Endometrial biopsy: Specimens were obtained via pipe suction curettage (Wallace) on 7 days after HCG injection or on day 5 after oocytes retraction. Tissue samples were washed thoroughly with sterile normal saline to remove excess blood and mucous. Specimens were frozen at − 80 °C for subsequent western blot or qRT-PCR analysis, or fixed in 10% formalin and embedded in paraffin for immunohistochemistry analysis or TUNEL staining. The demographic information was listed in Table 1.

**Table 1:** Basic information of participants

	Control group	Agonist group	Antagonist group
Sample size	15	11	15
Age (years)	31.3 ± 2.66	30.9 ± 2.91	31.0 ± 2.90
Body mass index	22.8 ± 3.46	21.3 ± 1.48	21.9 ± 3.06
Stimulation duration (day)	/	10.8 ± 1.47	9.33 ± 0.98*
Total Gn dose (IU)	/	2178.41 ± 465.8	2125 ± 527.23
Total Cetrorelex dose (mg)	/	/	1.3 ± 0.25
E2 on trigger day (pg/ml)	241.13 ± 69.55*	4180.27 ± 1282.49	4095.4 ± 1351.49
P on trigger day (ng/ml)	1.39 ± 0.52	1.38 ± 0.42	1.24 ± 0.36
LH on trigger day (U/L)	/	1.64 ± 0.92	2.21 ± 1.57
Number of oocytes retrieved	/	10.82 ± 3.06	10.2 ± 2.75
Endometrial thickness on biopsy day (cm)	0.86 ± 0.10	0.94 ± 0.19	0.92 ± 0.17
P on biopsy day (ng/ml)	18.7 ± 4.12	19.36 ± 4.56	19.97 ± 3.66

Data expressed as mean ± SD, and * *P* < .05

### Immunohistochemistry and immunofluorescence evaluation of endometrial tissue

Human endometrial tissues were fixed with formalin, embedded in paraffin, and sliced into 5-μm sections. Immunohistochemical or immunofluorescence approaches were conducted as previously described [15]. Briefly, the primary antibodies used are as follows: mouse anti-S100P monoclonal antibodies (MAB2957, R&D Systems, Minneapolis, MN, USA, 1:50 dilution), rabbit anti-cytokeratin monoclonal antibodies (ab181598, Abcam, UK, 1:100), rabbit anti-Vimentin monoclonal antibodies (ab92547, Abcam, UK, 1:100) and isotype antibody as negative control. Slides were then stained with secondary antibody (Cy3-conjugated anti-Mouse IgG, FITC-conjugated anti-Rabbit IgG or HRP-conjugated), followed by DAPI staining or hematoxylin counterstaining for immunofluorescence and immunohistochemistry, respectively. Slide images were visualized using a laser confocal microscope (Leica SP8, Germany) or an upright microscope (Nikon Inc., Japan). Densitometric analysis was conducted to compare the expression level of proteins using Image J (Version 1.5.1; NIH, Bethesda, MD, USA).

### Protein expression analysis by western blot

Protein was extracted from endometrial tissue samples or cultured cells using RIPA buffer containing protease inhibitors (89,900, Thermo, USA). Equal amounts of 30 μg protein were separated by 12% sodium dodecyl sulfate–polyacrylamide gel electrophoresis (SDS-PAGE) and transferred to polyvinylidene difluoride (PVDF) membranes (Millipore, Billerica, MA, United States). Then, the membranes were incubated with the following specific primary antibodies: S100P (ab133554, Abcam, UK, 1:1000), Bax (5023 T, Cell Signaling Technology, 1:1000), Bcl-2 (4223S, Cell Signaling Technology, 1:1000), HOXA10 (ab191470, Abcam, UK, 1:1000), GAPDH (5174, Cell Signaling Technology, 1:1000). Afterwards, the membranes were incubated with the corresponding horseradish peroxidase-conjugated secondary antibodies (Beyotime Biotechnology, China, 1:1000). Finally, protein bands were detected by enhanced chemiluminescence (ECL) according to the manufacturer’s (Millipore) instructions.

### RNA isolation and real time PCR

Total RNA was extracted from primary human endometrial epithelial cells using Trizol (Invitrogen), and RNA was reverse-transcribed using a PrimeScript™ RT reagent Kit (Takara, RR037A), as described in the product manual. Real time PCR was performed with TB Green® Premix Ex Taq™ II kit (Takara, RR820A). The primers used the same as previously described [16]. Each analysis was performed in triplicate.

### TUNEL assay

Endometrial tissue apoptosis was assayed using the Cell Apoptosis Detection Kit–POD (cat#G1507, Servicebio, China), according to manufacturer’s instructions. In brief, paraffin-embedded tissue sections, were heated at 60 °C, washed in xylene, and rehydrated through a graded series of ethanol and water. Tissue sections were then incubated for 30 min at room temperature with a Proteinase K solution. Slides were rinsed twice with PBS then 50 μl of TUNEL reaction mixture was added to each section and incubated for 60 min at 37 °C in a humidified atmosphere. Labeling solution (without terminal transferase) instead of TUNEL reaction mixture was added to control slides as a negative staining control. Finally, each sample incubated with 50 μl Converter-POD in a humidified chamber for 30 min, followed by addition of 100 μl DAB substrate. Slides were analyzed under a light microscope.

### Detection of apoptosis by flow cytometry

Flow cytometry was used to analyze the influence of S100P downregulation on GnRH-ant-induced apoptosis of Ishikawa cells. Treated cells were exposed to 25 μM H_2_O_2_ for 24 h and then resuspended in Binding Buffer at 1–5 × 10^6^ cells/ml. Each sample was stained for 15 min at room temperature with 3 μl of fluorochrome-conjugated Annexin V and 5 μl of 7-AAD was added to 100 μl of the cell suspension. The cells were then analyzed using flow cytometry (CytoFLEX, Beckman-Coulter, Germany).

### Cell line culture and treatments

Ishikawa cells, a human endometrial epithelial cell line, were maintained in DMEM/F12 (Gibco, USA) supplemented with 10% fetal bovine serum (Gibco, USA), at 37 °C in a humid atmosphere with 5% CO_2_. Cells were treated with 1 × 10^− 5^ M GnRH-antagonist, and culture supernatant was collected every 12 h over two consecutive days. An ELISA kit (CSB-E14080h, China) was used to evaluate levels of S100P secretory protein in the supernatants, and supernatants from untreated cells were loaded as a control. S100P overexpression and knockdown with siRNA were performed using Lipofectamine 3000 reagent (Lipofectamine 3000, L3000008, Thermofisher, USA), as previously described [16]. The negative control and S100P-siRNA oligonucleotides (sense: AAGGAUGCCGUGGAUAAAUdTdT; antisense: AUUUAUCCACGGCAUCCUUdTdT) were synthesized by Genepharma (Shanghai, China).

### Statistical analysis

Statistical analysis was performed using SPSS software (Version 23, IBM Corp., Armonk, NY, USA). A two-tailed Student’s t-test or one-way analysis followed by Tukey’s multiple comparisons post hoc test were used to determine statistical significance. Continuous variables are expressed as mean ± SD. Enumeration data are expressed as percentage values. A *p*-value of *P* < 0.05 was considered to be statistically significant.

## Results

### Basic information of the participants

There was no difference in demographic information about age and BMI among the three groups. The serum estrogen level on trigger day in control group was significantly lower than in the other two ovulation groups. The stimulation duration of GnRH-ant group was shorter than GnRH-a group. Other indexes about ovulation and hormonal levels were not different among these groups.

### Increased apoptosis is detected in endometrial tissue of patients treated with GnRH-ant protocol

**Fig. 1:**
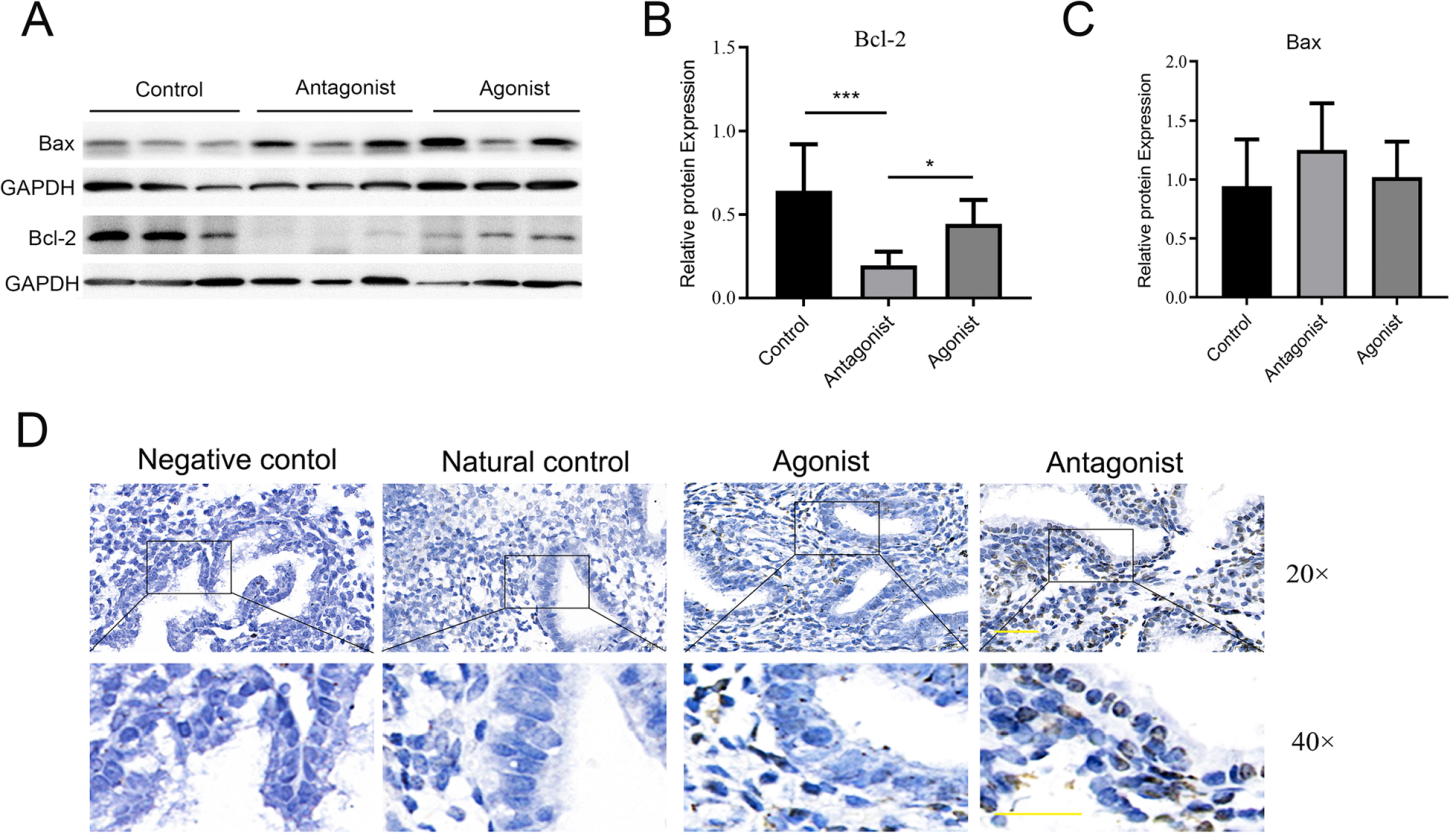
Apoptosis is increased in the mid-secretory phase endometrium of participants from GnRH-ant group. **A** Representative western blot showing protein expression of Bax and Bcl-2 in the endometrium of the natural control, GnRH-ant and GnRH-a groups. **B** and **C** Statistical analysis of Bcl-2 and Bax protein expression in the endometrium of the natural control, GnRH-ant and GnRH-a groups. **D** TUNEL assay evaluation of apoptosis in the endometrium of the different groups. Negative control used PBS to substitute for the TUNEL reagent. The scale bar represents 50um. *P < .05, *** *P*<.001

The expression of Bcl-2 protein in the GnRH-ant group was siginificantly lower than in the control or GnRH-a group, as demonstrated by western blot (Fig. 1A, B). The expression of Bax in the GnRH-ant group was increased but did not reach statistical significance (Fig. 1A, C). TUNEL assay was employed to detect apoptosis in endometrial tissue among patients undergoing different IVF protocols at day LH + 7. Apoptosis was higher in the endometrial epithelium of patients in the GnRH-ant group than in the GnRH-a or the control group, as demonstrated by TUNEL assay (Fig. 1D).

### S100P is significantly down-regulated in the endometrium of patients treated with the GnRH-ant protocol

**Fig. 2:**
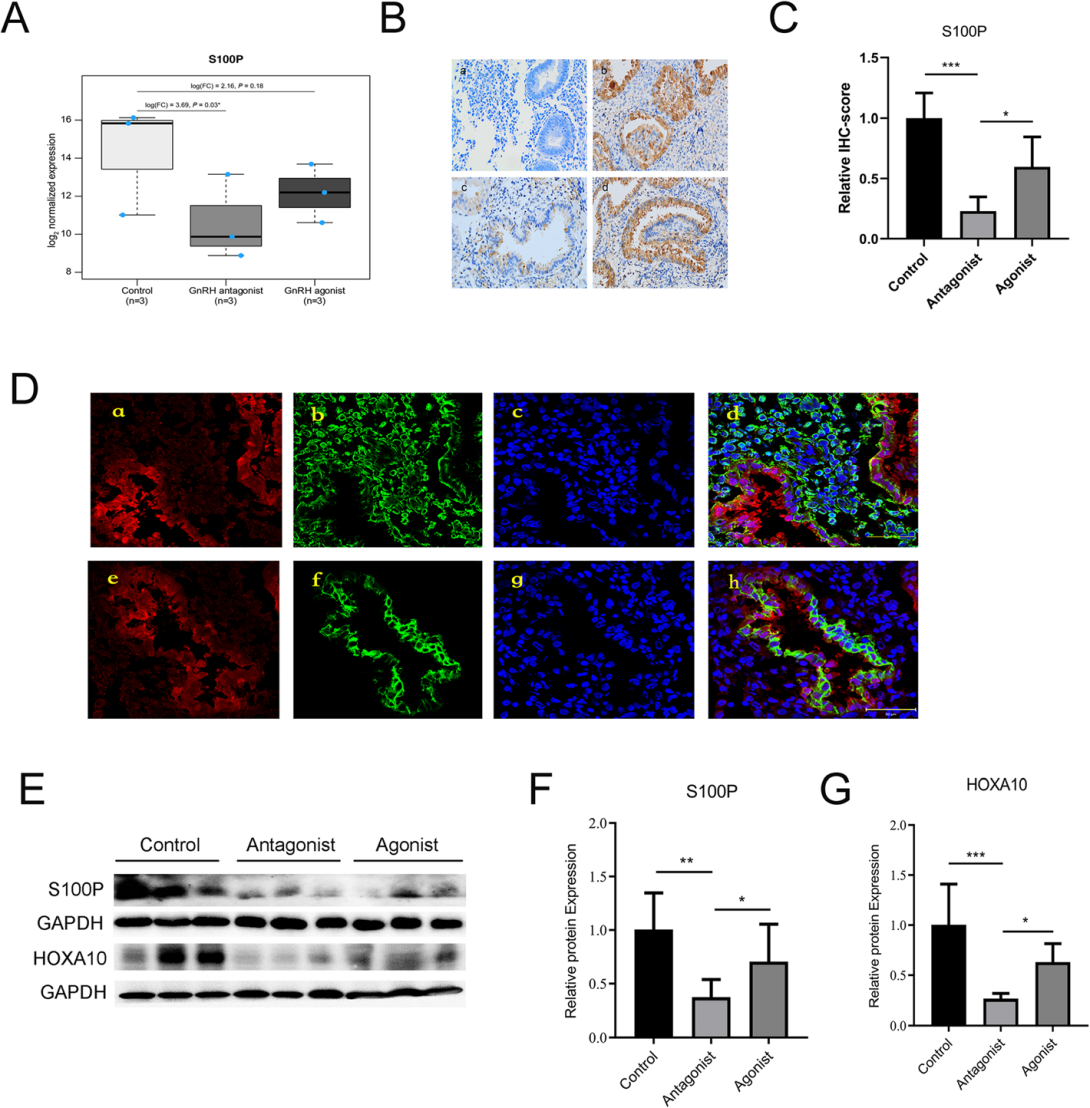
S100P is down-regulated in the mid-secretory phase endometrium of participants from GnRH-ant group. **A** S100P gene expression in different groups from microarray data. **B** Immunohistochemical detection of S100P in the endometrium from the different groups, magnified in 200 times and the scale bar represents 50um. a. Negative control, b. Natural cycle control, c. GnRH antagonist group, d. GnRH agonist group; quantified in (**C**). **D** Location of S100P in endometrium by co-localization with vimentin and keratin. Red: S100P; Green: vimentin (b) and cytokeratin (f); Blue: nucleus. The scale bar represents 50um. **E** Western blot results of S100P and HOXA10 protein expression in endometrium from the natural cycle, GnRH-ant and GnRH-a groups. **F** Quantification of S100P protein expression. **G** Quantification of HOXA10 protein expression. *P < .05, **P<.01, ***P<.001

We evaluated S100P expression by microarray from a previous cohort [9], and found that the expression of S100P in endometrial tissue during the mid-secretory phase was significantly lower in the GnRH-ant group compared to the normal control (Fig. 2A). Immunohistochemistry staining and western blot analysis showed that S100P expression was significantly decreased in the endometrium of the GnRH-ant group when compared to the normal control or the GnRH-a group (Fig. 2B, C, E and F). Co-localization analysis revealed co-localization of S100P and cytokeratin, but no co-localization was found between S100P and vimentin (Fig. 2 D). The expression of HOXA10, a biomarker of endometrial receptivity was also significantly decreased in GnRH-ant group compared to the normal control or GnRH-a group. (Fig. 2E, G).

### Knockdown of S100P induces apoptosis in endometrial epithelial cells

**Fig. 3:**
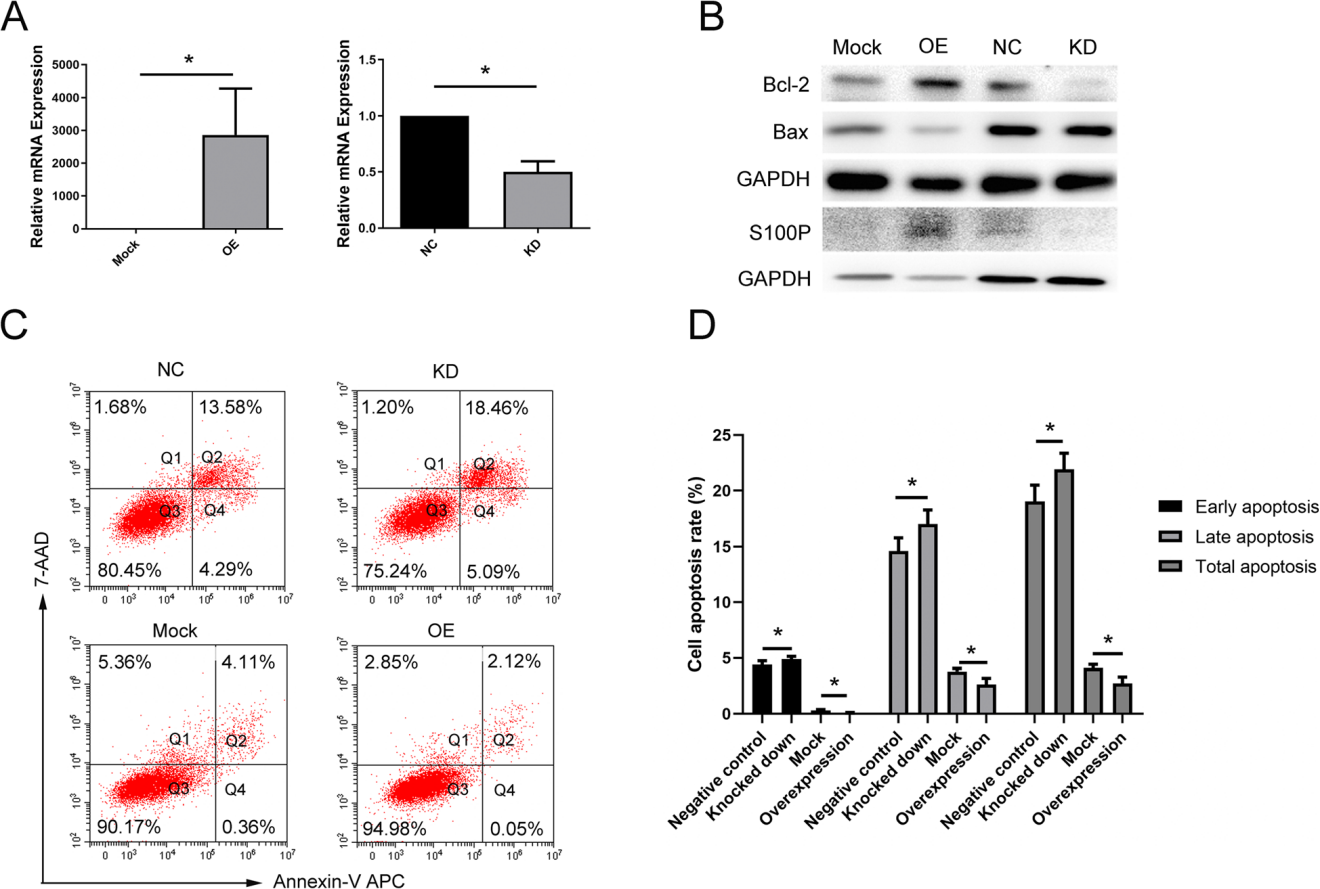
Knockdown of S100P induces apoptosis in endometrial epithelial cells. **A** Overexpression and knockdown of S100P mRNA was verified by Realtime PCR. **B** Overexpression and knockdown of S100P protein was verified and expression of Bcl-2 and Bax protein was analyzed by western blot. **C** Apoptosis rate was evaluated by flow cytometry after overexpression and knockdown of S100P in Ishikawa cells; results are quantified and analyzed in (**D**). NC: negative control using scramble sequence; KD: S100P knockdown; Mock: mock control transfected with empty plasmid vector; OE: S100P overexpression; *P < .05

We evaluated the effects that siRNA-mediated knockdown or lentiviral-mediated overexpression of S100P had on Ishikawa cells, which are commonly used as a model to study endometrial receptivity. The expression of S100P mRNA was inhibited by more than 50% by the siRNA, and was overexpressed by more than 1000 times by the lentiviral overexpression vector, when compared to the scramble sequence control or the empty vector control, respectively (Fig. 3A). Western blot demonstrated that S100P protein expression was significantly higher in the overexpression group and significantly lower in the knockdown group (Fig. 3B). We then examined apoptosis rates in Ishikawa cells after S100P overexpression or knockdown. Flow cytometry analysis showed that knockdown of S100P induced apoptosis, while overexpression of S100P reduced apoptosis (Fig. 3C, D). Additionally, expression of the anti-apoptotic protein Bcl-2 was decreased, and expression of the pro-apoptotic protein Bax was increased, in Ishikawa cells after S100P knockdown. In contrast, overexpression of S100P in Ishikawa cells decreased Bcl-2 expression and increased Bax expression (Fig. 3B).

### Increased S100P expression reduces the apoptosis induced by GnRH antagonist in endometrial epithelial cells

**Fig. 4:**
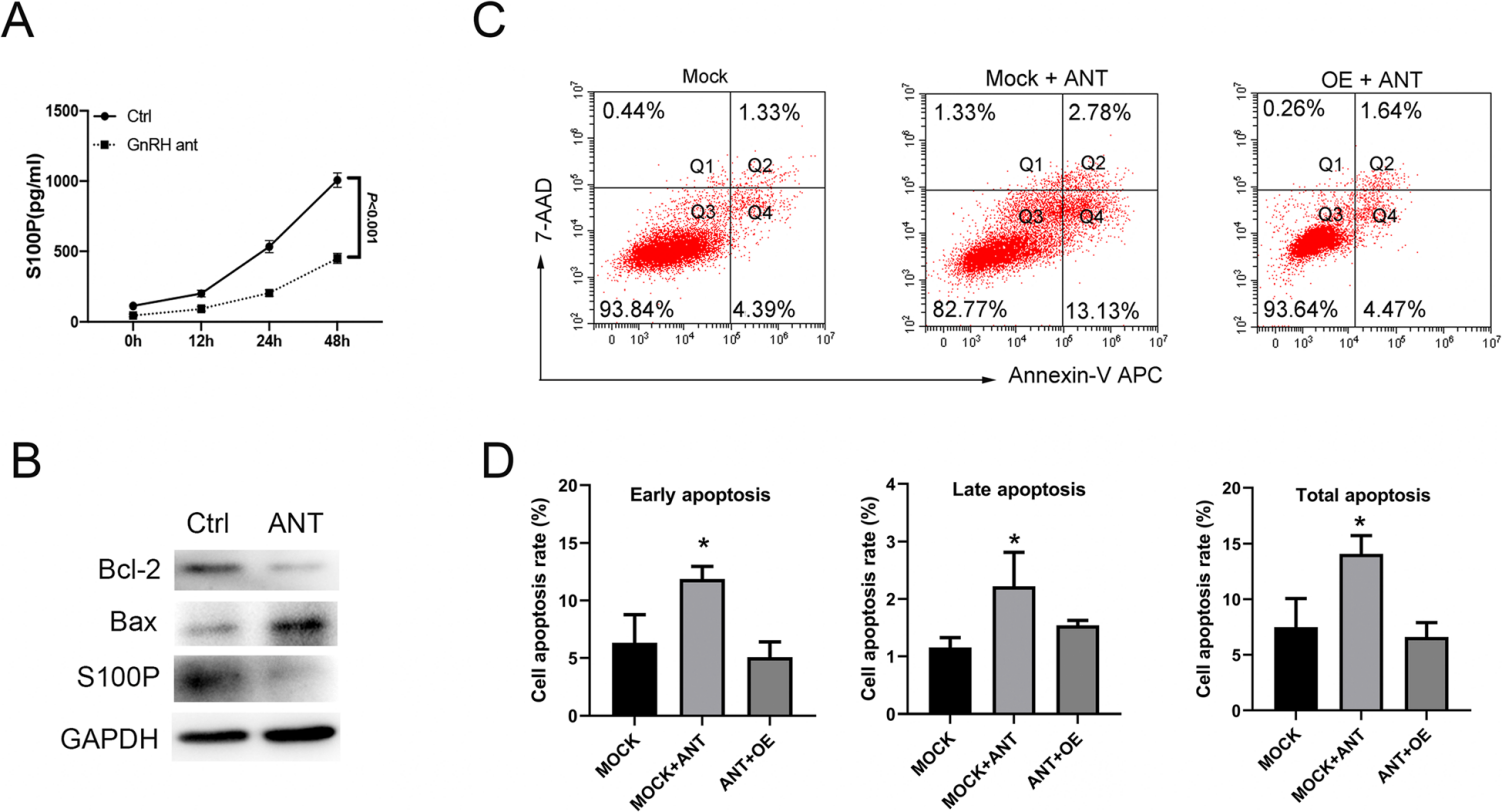
GnRH antagonist-induced apoptosis in endometrial epithelial cells is rescued by S100P overexpression. **A** S100P concentration in the cell culture supernatant after GnRH-ant treatment, as determined by ELISA. **B** Protein expression of S100P, Bcl-2 and Bax in cultured cells after GnRH-ant treatment, as determined by western blot. **C** Flow cytometry analysis of apoptosis rate among different treatment groups; quantified and analyzed in (**D**). Mock: mock control transfected with empty plasmid vector; ANT: cells exposed to the GnRH antagonist- Cetrorelix; OE: S100P overexpression; *P < .05

We determined the effects of GnRH antagonist on endometrial epithelial cells in vitro by adding Cetrorelix, a GnRH antagonist, to the cell culture medium. After 48 h, cell culture supernatant was collected to evaluate the expression of secreted S100P, and cells were harvested to evaluate apoptosis. Levels of both extracellular and intracellular S100P were significantly decreased after exposure to Cetrorelix, as demonstrated by ELISA evaluation and western blot analysis, respectively (Fig. 4A, B). Expression of the anti-apoptotic protein Bcl-2 was decreased and expression of the pro-apoptotic protein Bax was increased in Ishikawa cells after exposure to Cetrorelix (Fig. 4B). Likewise, flow cytometry analysis showed an increased apoptosis rate in Ishikawa cells after exposure to Cetrorelix. However, no increase in apoptosis rate was observed in the S100P overexpressing cells treated with Cetrorelix (Fig. 4C, D).

## Discussion

The establishment of endometrial receptivity is a prerequisite for successful embryo implantation. Endometrial receptivity involves a series of complex and dynamic changes in the endometrium, and endometrial damage and dysplasia can lead to inadequate endometrial receptivity. Appropriate endometrial epithelial cell apoptosis is of great importance in establishing endometrial receptivity [17]. In the present study, we found that the expression of the anti-apoptotic protein Bcl-2 was significantly decreased in endometrial tissue of patients in the GnRH-ant group when compared to the control or the GnRH-a group. We also observed elevated apoptosis in the endometrial epithelium of patients treated with the GnRH-ant protocol. The abnormal elevated apoptosis in the endometrium of patients undergoing the GnRH-ant protocol should be unfavorable to endometrial receptivity.

S100P is a potential biomarker for endometrial receptivity. We have previously reported that S100P in the endometrium is expressed approximately 100 times higher in the mid-secretory phase than in other phases of the menstrual cycle in normal fertile women [18, 19]. Additionally, the expression of S100P is significantly increased in the trophoblast during the early stage of placental formation [20, 21], and is increased in endometrial cells after co-culture with trophoblast cells [22]. These results suggest that S100P play a pivotal role in the establishment of endometrial receptivity, and also be involved in interactions at the maternal-fetal interface. In the present study, we observed that S100P was significantly decreased in the endometrium of patients in the GnRH-ant group compared to the control or the GnRH-a group. The expression of S100P was lower in GnRH-a group than in control group, but with no significant difference. These results were in line with previous reports regarding endometrial receptivity among patients undergoing different IVF protocols, in which it was observed that endometrial receptivity was reduced during ovulation cycles, including in both GnRH-a and GnRH-ant cycles, and was lower in patients undergoing GnRH-ant protocol cycles than in those undergoing the conventional GnRH-a long protocol cycles [23, 24]. This suggested that a reduction in S100P might act as a mechanism to mediate the decreased endometrial receptivity that can result from the GnRH-ant protocol. The concurrent decrease of HOXA10, a classical biomarker of endometrial receptivity, further confirmed the decreased endometrial receptivity in the GnRH-ant group.

S100P influences cell proliferation, invasion and motility, angiogenesis, cytoskeletal interactions, and inflammation [25]. S100P has also been reported to reduce apoptosis to promote cancer progression [14, 26]. In the present study, we verified the expression of S100P was mainly distributed in the endometrial epithelial cells, where was in accord with the elevated apoptosis in the GnRH-ant group showed by TUNEL assay. Here, we used Ishikawa cells as an in vitro model to evaluate the impact of changes in expression of S100P on the effects of GnRH-ant treatment on endometrial epithelial cells. Ishikawa cells are an endometrial epithelial cell line derived from a moderate differentiated endometrial adenocarcinoma, and are commonly used as an in vitro model for endometrial epithelial cells [27]. S100P promotes the proliferation, invasion, and migration of endometrial cells and trophoblast cells, and is known to promote the progression of endometrial cancer and facilitate embryo implantation [21, 25]. In this study, we discovered that knockdown of S100P promoted apoptosis in endometrial epithelial cells, while overexpression of S100P reduced apoptosis induced by GnRH-ant treatment. Hongfei Jiang et al. reported the same phenomenon in HEC-1 cells [28], another endometrial epithelial cell model; this reinforces our conclusion that down-regulation of S100P induces endometrial epithelial cell apoptosis.

It has been reported that knocking down S100P promotes cell apoptosis by regulating expression of apoptosis related factors, including Bax, caspase-3, FASLG, DAPK1, CTNNB1, and Bcl-2 [14, 26, 29, 30]. In this study, the anti-apoptotic protein Bcl-2 was decreased following knockdown of S100P, and was increased when S100P was overexpressed. The pro-apoptotic factor Bax was significantly decreased after overexpression of S100P, although it was unchanged following S100P knockdown. This indicated that the up-regulation of Bcl-2 and the down-regulation of Bax may mediate the inhibitory effects of elevated S100P on apoptosis in endometrial epithelial cells. However, the decrease of S100P may promote apoptosis mainly mediated by down-regulation of Bcl-2 in endometrial epithelial cells.

Our in vitro experiments showed that S100P decreased both in the cell culture supernatant and in the cultured cells themselves following exposure to GnRH-ant. This suggested that the decreased S100P in the endometrium of the GnRH-ant group may be directly caused by the GnRH-ant treatment. Our results also identified an increase in apoptosis rate in the endometrial epithelial cells after exposure to GnRH-ant, characterized by decreased Bcl-2 expression and increased Bax expression. These results were in agreement with a report that Cetrorelix reduced the expression of the anti-apoptotic proteins Bcl-2 and Bcl-x(L) in myeloma cells, and thus induced apoptosis [31]. However, Zhao XJ et al. found that GnRH-ant treatment reduced chemotherapy-induced apoptosis in granulosa cells triggered by cyclophosphamide treatment, but that could be a phenomenon specific to the treatment with cyclophosphamide [32].

We also found that exposure to GnRH-ant did not increase the apoptosis rate in the S100P overexpressing Ishikawa cells. This demonstrated that S100P overexpression can rescue the apoptosis induced by GnRH-ant treatment. Furthermore, these results suggested that although many identified factors, such as serum hCG, estrogen or progesterone levels, may influence the endometrial receptivity in ovulation cycles, the down-regulation of S100P caused by GnRH-ant may be a mechanism by which endometrial receptivity is reduced in the GnRH-ant protocol, and indicate that S100P may be a potential target to intervene in reduced endometrial receptivity. To the best of our knowledge, this is the first report of suggesting that GnRH-ant induces apoptosis through down-regulation of S100P in human endometrial epithelial cells.

This study is not without its limitations. One of the limitations is that we employed Ishikawa cell as a model to substitute for primary endometrial epithelial cells. Using primary epithelial cells may have provided a more suitable model, but would have introduced significant difficulties regarding the challenging culture of primary cells and the possibility that substantial differences between primary cell cultures could limit reproducibility of results.

## Conclusions

In summary, we found that increased apoptosis and down-regulation of S100P in the mid-secretory phase endometrium of patients undergoing the GnRH-ant protocol, which may contribute to reduced endometrial receptivity. In addition, in vitro exposure to GnRH-ant and knockdown of S100P by siRNA induced apoptosis of endometrial epithelial cells mainly through the Bcl-2 pathway. Furthermore, S100P overexpression rescued the GnRH-ant induced apoptosis in endometrial epithelial cells. Although further studies are needed to more clearly illuminate the mechanism by which GnRH-ant impacts S100P, our observations suggest that S100P could be a new therapeutic target to improve endometrial receptivity and increase the success of pregnancy following the GnRH-ant protocol, thereby reducing implantation failure rates in IVF.

## Data Availability

The datasets used and/or analyzed in this study are available from the corresponding author on reasonable request.

## Abbreviations

GnRH: Gonadotropin releasing hormone
GnRH-ant: Gonadotropin releasing hormone antagonist
GnRH-a: Gonadotropin releasing hormone agonist
IVF: In vitro fertilization
S100P: S100 calcium protein P
